# Iron deficiency responses in rice roots

**DOI:** 10.1186/s12284-014-0027-0

**Published:** 2014-10-07

**Authors:** Takanori Kobayashi, Reiko Nakanishi Itai, Naoko K. Nishizawa

**Affiliations:** Japan Science and Technology Agency, PRESTO, 4-1-8 Honcho, Kawaguchi, Saitama, 332-0012 Japan; Research Institute for Bioresources and Biotechnology, Ishikawa Prefectural University, 1-308 Suematsu, Nonoichi, Ishikawa, 921-8836 Japan; Graduate School of Agricultural and Life Sciences, The University of Tokyo, 1-1-1 Yayoi, Bunkyo-ku, Tokyo, 113-8657 Japan

**Keywords:** Gene regulation, Iron deficiency response, Mugineic acid family phytosiderophores, Transcription factor, Transporter

## Abstract

Iron (Fe) is an essential element for most living organisms. To acquire sparingly soluble Fe from the rhizosphere, rice roots rely on two Fe acquisition pathways. The first of these pathways involves Fe(III) chelators specific to graminaceous plants, the mugineic acid family phytosiderophores, and the second involves absorption of Fe^2+^. Key components in this response include enzymes involved in the biosynthesis of deoxymugineic acid (OsNAS1, OsNAS2, OsNAAT1, and OsDMAS1), the deoxymugineic acid efflux transporter (TOM1), the Fe(III)-deoxymugineic acid transporter (OsYSL15), and Fe^2+^ transporters (OsIRT1, OsIRT2, and OsNRAMP1). In whole roots, these proteins are expressed in a coordinated manner with strong transcriptional induction in response to Fe deficiency. Radial transport of Fe to xylem and phloem is also mediated by the mugineic acid family phytosiderophores, as well as other chelators and their transporters, including Fe(II)-nicotianamine transporter (OsYSL2), phenolics efflux transporters (PEZ1 and PEZ2), and citrate efflux transporter (OsFRDL1). Among these, OsYSL2 is strongly induced under conditions of Fe deficiency. Both transcriptional induction and potential feedback repression mediate the expressional regulation of the genes involved in Fe uptake and translocation in response to Fe deficiency. The transcription factors IDEF1, IDEF2, and OsIRO2 are responsible for transcriptional induction, whereas the ubiquitin ligases OsHRZ1 and OsHRZ2, as well as the transcription factors OsIRO3 and OsbHLH133, are thought to mediate negative regulation. Furthermore, IDEF1 and OsHRZs bind Fe and other metals, and are therefore candidate Fe sensors. The interacting functions of these regulators are thought to fine tune the expression of proteins involved in Fe uptake and translocation.

**Electronic supplementary material:**

The online version of this article (doi:10.1186/s12284-014-0027-0) contains supplementary material, which is available to authorized users.

## Review

Iron (Fe) is an essential element for most living organisms, including all animals and plants. However, despite its high abundance in the Earth's crust, Fe is only slightly soluble in the soil under aerobic conditions, especially in alkaline calcareous soils (Marschner [1995]). To prevent Fe deficiency, rice plants possess a dual mechanism in which ferric iron [Fe(III)] is taken up as a complex with iron-chelating compounds, and ferrous ion (Fe^2+^) is taken up directly (Takagi [1976]; Römheld and Marschner [1986]; Ishimaru et al. [2006]). Excessive Fe uptake is highly deleterious, as it catalyzes the generation of reactive oxygen species. Thus, to optimize Fe uptake, genes responsible for Fe uptake and translocation are transcriptionally induced in response to low Fe availability, and repressed when sufficient Fe has been absorbed. Recent advances in molecular biology and bioinformatics linked to rice genome sequence data have markedly advanced our understanding of Fe deficiency responses at the molecular level. In this review, we summarize rice root responses to Fe deficiency, which consist of Fe uptake from the rhizosphere, Fe translocation to the xylem and phloem, and their expressional modulation by positive and negative regulators.

In spite of these root responses, rice is highly susceptible to Fe deficiency (Mori et al. [1991]). Insufficient Fe causes the plant disease Fe chlorosis, which is characterized by yellowing of new leaves and severely impacts grain yield. Rice is also a characteristic crop which contains low concentration of Fe in polished seeds, thus is disadvantageous as a major Fe supply in human diet. Understanding of the molecular components of Fe uptake and translocation and their regulation has paved the way to develop crops that are tolerant to Fe deficiency, both to improve food and biomass production, as well as to develop Fe-rich crops for improved human nutrition (reviewed in Kobayashi and Nishizawa [2012]; Bashir et al. [2013]; Masuda et al. [2013]).

### Iron uptake from the rhizosphere

**Figure 1:**
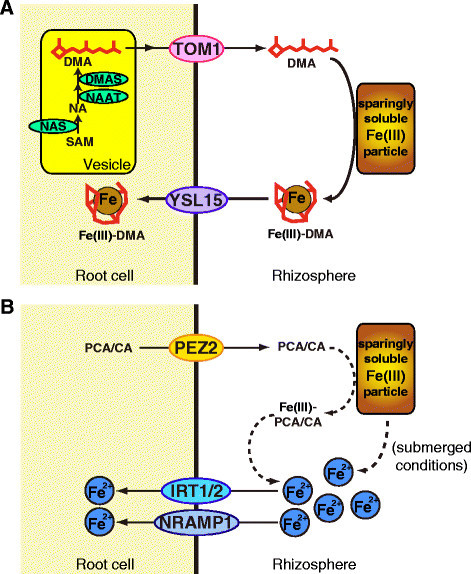
**Fe acquisition systems in rice roots. A)** Strategy II system. **B)** Partial Strategy I system. Ovals represent transporters and enzymes that play central roles in Fe uptake from the rhizosphere. All the indicated transporters and enzymes except PEZ2 are strongly induced in response to Fe deficiency. Broken lines indicate putative pathways. DMA, 2′-deoxymugineic acid, NA, nicotianamine, SAM, *S*-adenosyl-L-methionine; PCA, protocatechuic acid; CA, caffeic acid.

Takagi ([1976]) was the first to describe natural Fe(III) chelators secreted from the roots of graminaceous plants, which were designated as mugineic acid family phytosiderophores (MAs). The biosynthesis and secretion of MAs are unique to graminaceous plants. Römheld and Marschner ([1986]) classified plant Fe uptake mechanisms into two basic strategies: iron absorption through reduction (Strategy I) in non-graminaceous plants and iron absorption through chelation (Strategy II) in graminaceous plants. Since then, the molecular components of both strategies have been identified (Kobayashi and Nishizawa [2012]; Rodríguez-Celma et al. [2013]; Fourcroy et al. [2014]; Schmid et al. [2014]). Rice, being a graminaceous species, utilizes Strategy II (Figure 1A), but also possesses a partial Strategy I system (Figure 1B) (Ishimaru et al. [2006]).

Among the nine types of MAs identified, young rice plants exclusively synthesize and secrete 2′-deoxymugineic acid (DMA) (Nakanishi et al. [2000]). All MAs share a conserved biosynthetic pathway from methionine to DMA (Mori and Nishizawa [1987]; Kawai et al. [1988]; Shojima et al. [1990]), which is mediated by nicotianamine synthase (NAS), nicotianamine aminotransferase (NAAT), and deoxymugineic acid synthase (DMAS) (Higuchi et al. [1999]; Takahashi et al. [1999]; Bashir et al. [2006]). In rice, the enzymes participating in this pathway include OsNAS1, OsNAS2, OsNAAT1, and OsDMAS1 (Table 1) (Higuchi et al. [2001]; Inoue et al. [2003], [2008]; Bashir et al. [2006]; Cheng et al. [2007]). Genes encoding enzymes involved in the methionine cycle are also transcriptionally induced to ensure a sufficient supply of methionine for DMA biosynthesis (Kobayashi et al. [2005]; Itai et al. [2013]).

**Table 1:** **Rice genes responsible for Fe uptake and translocation, and their expression patterns under Fe deficiency**

		Fe deficiency response					Effects of regulators					
Gene name	Function	Whole root		Microdissected			IDEF1		IDEF2	IRO2	HRZ1/2	
		24 h	7d	7d Ep	7d Co	7d VB	1d	2-7d	7d	6d	Cont.	7d
**DMA biosynthesis for Fe(III)-DMA uptake/translocation**												
*OsNAS1*	Nicotianamine synthase	↑	↑↑	↑	↑↑	↑↑	↑	(↓)^c^	→	↑	↓↓	→
*OsNAS2*	Nicotianamine synthase	↑	↑↑	↑↑	↑↑	↑↑	↑	(↓)^c^	→	↑	↓↓	→
*OsNAS3*	Nicotianamine synthase	→	↑	↑	↑↑	↑	↑	↑	↓	↓	↓	↓
*OsNAAT1*	Nicotianamine aminotransferase	↑	↑↑	↑↑	↑↑	↑↑	(↓)^c^	(↓)^c^	→	↑	↓	→
*OsDMAS1*	Deoxymugineic acid synthase	↑	↑↑	↑↑	↑↑	↑↑	↑	(↓)^c^	→	↑	↓	→
**Transporters for Fe(III)-DMA uptake/translocation**												
*TOM1*	DMA efflux transporter	↑	↑↑	↑↑	↑↑	↑↑	↑?^d^	(↓)^c^	(↓)	↑^e^	↓↓	→
*OsYSL15*	Fe(III)-DMA transporter	↑	↑↑	↑↑	↑↑	↑↑	↑	→	→	↑	↓↓	→
*OsYSL16*	Fe(III)-DMA transporter^a^	→	↑	(↑)	(↓)	→	→	→	→	→	→	→
**Methionine cycle for Fe(III)-DMA uptake/translocation**												
*OsSAMS1*	*S* -adenosyl-L-methionine synthetase	→	(↑)	→	→	→	→	→	→	→	→	→
*OsSAMS2*	*S* -adenosyl-L-methionine synthetase	(↑)	↑	↑	↑↑	↑	(↑)	→	→	→	(↓)	→
*MTN*	Methylthioadenosine/*S* -adenosyl homocysteine nucleosidase	(↑)	↑	↑	↑↑	↑	(↑)	→	→	↑	↓	→
*OsMTK1*	Methylthioribose kinase	↑	↑^b^	↑↑	↑↑	↑	→	(↓)	→	→	↓	→
*OsMTK2*	Methylthioribose kinase	↑	↑^b^	↑↑	↑↑	↑	→	(↓)	→	→	→	→
*OsIDI2*	Methylthioribose-1-phosphate isomerase	↑	↑	↑↑	↑↑	↑	→	↓	→	↑	↓	→
*DEP*	Methylthioribulose-1-phosphate dehydratase-enolase-phosphatase	↑	↑	↑↑	↑↑	↑↑	(↑)	↓_c_	(↓)	↑	↓	→
*OsIDI1/OsARD2*	Acireductone dioxygenase	↑	↑	↑↑	↑↑	↑	↑	→	→	↑	↓	→
*OsIDI1L/OsARD1*	Acireductone dioxygenase	↑	↓	(↑)	(↓)	↓	(↑)	↑	↓	→	(↓)	(↓)
*OsIDI4*	Aminotransferase catalyzing the synthesis of methionine?	↑	↑	↑↑	↑↑	↑↑	↑	(↓)	→	↑	↓	→
*OsAPT1*	Adenine phosphoribosyltransferase	↑	↑	↑↑	↑↑	↑	(↑)	(↓)	→	↑	(↓)	→
*PRPPS*	Phosphoribosyl pyrophosphate synthetase	↑	↑	↑↑	↑↑	↑	(↑)	↓^c^	(↓)	(↑)	(↓)	→
*RPI*	Ribose 5-phosphate isomerase	↑	↑	↑↑	↑↑	↑	(↑)	→	→	→	→	→
*FDH*	Formate dehydrogenase	↑	↑	↑↑	↑↑	↑	↑	↓^c^	→	↑	↓	→
**Transporters for ferrous Fe uptake/translocation**												
*OsIRT1*	Ferrous Fe transporter	→	↑↑	↑	↑↑	↑↑	↑	↑	→	→	↓	→
*OsIRT2*	Ferrous Fe transporter	↑	↑↑	↑↑	↑↑	↑↑	↑	↑	→	n.d.	↓	→
*OsNRAMP1*	Ferrous Fe transporter	↑	↑↑	↑↑	↑↑	↑↑	↑	↑	→	→	↓	→
*OsNRAMP5*	Ferrous Fe/manganese/cadmium transporter	(↓)	↑	(↑)	↑	(↑)	(↓)	→	→	→	↓	(↓)
*PEZ2*	Phenolics efflux transporter	→	→	(↑)	↑	↑	→	(↓)	→	→	→	→
**Transporters for Fe translocation**												
*OsYSL2*	Fe(II)/manganese(II)-NA transporter	↑	↑↑	↑↑	↑↑	↑↑	↑	(↑)	↑↑	→^e^	↓↓	↓↓
*ENA1*	NA efflux transporter	→	↑	↑	↑↑	↑↑	→	(↑)	(↓)	→	↓	(↓)
*ENA2*	NA efflux transporter	→	(↑)	→	(↑)	(↑)	→	→	→	n.d.	→	→
*OsFRDL1*	Citrate efflux transporter	→	→	↓	(↑)	→	→	→	→	→	→	→
*PEZ1*	Phenolics efflux transporter	→	(↓)	→	(↓)	↑	→	→	↓	n.d.	→	→
**Transporters for subcellular Fe sequestration**												
*OsVIT1*	Fe transporter into vacuole	→	↓	→	→	→	→	→	→	→	→	→
*OsVIT2*	Fe transporter into vacuole	↓	↓↓	→	↓	↓↓	→	→	→	→	(↑)	→
*MIT*	Fe transporter into mitochondria	→	↓	(↑)	→	→	→	→	→	n.d.	→	→
**Gene regulation in response to Fe deficiency**												
*IDEF1*	Positive transcriptional regulator	→	→	↓	(↓)	↓	-	-	→	→	→	→
*IDEF2*	Positive transcriptional regulator	→	→	→	→	→	→	(↑)	-	→	→	→
*OsIRO2*	Positive transcriptional regulator	↑	↑↑	↑↑	↑↑	↑↑	↑	↑	(↓)	-	↓	→
*OsIRO3*	Transcriptional regulator (negative?)	↑	↑↑	↑	↑↑	↑↑	↑	↑	(↑)	→	↓	(↓)
*OsbHLH133*	Negative transcriptional regulator	↑	↑↑	n.d.	n.d.	n.d.	n.d.	n.d.	n.d.	n.d.	n.d.	n.d.
*OsHRZ1*	Negative regulator/ubiquitin ligase	↑	↑	↑↑	↑↑	↑	(↑)	(↑)	→	n.d.	-	-
*OsHRZ2*	Negative regulator/ubiquitin ligase	↑	↑	↑	↑↑	(↑)	→	→	→	→	-	-
*OsHORZ1*	Positive regulator?	→	(↑)	→	→	→	→	→	(↓)	→	→	→
*IBP1.1*	IDEF1 protector/trypsin inhibitor	↑	(↑)	(↓)	↓	↓↓	↑	(↑)	↓	→	→	→
*IBP1.2*	IDEF1 protector?/trypsin inhibitor	↑	↑	→	↓	↓↓	↑	↑	↓	→	→	→
*OsRMC*	Positive regulator?/receptor-like protein	↑	↓	→	↑	→	→	(↓)^c^	(↑)	→	(↓)	(↓)

Functions indicated with a question mark have not been confirmed. Arrows indicate expressional responses: ↑↑, strongly upregulated; ↑, upregulated; (↑), weakly upregulated; →, no significant change; (↓), weakly downregulated; ↓, downregulated; ↓↓, strongly downregulated; n.d., not determined because of the lack of corresponding probe in the microarray. Arrows in boldface indicate expression confirmed by quantitative RT-PCR and/or Northern blotting experiments. The remaining expression data are based on microarray results as follows: *Whole root 24 h*, root under 24-h Fe deficiency (Itai et al. [2013]); *Whole root 7d*, root under 7-d Fe deficiency (Ogo et al. [2008]); *Microdissected 7d Ep, 7d Co, and 7d VB*, rice root segments (Ep, epidermis plus exodermis; Co, cortex; VB, vascular bundle) under 7-d Fe deficiency (Ogo et al. [2014]); *IDEF1 1d and 2-7d*, roots from an *IDEF1* induction line *vs.* non-transformant under 1-d and 4-d Fe deficiency, respectively (Kobayashi et al. [2009]); *IDEF2 7d*, roots from an *IDEF2* knockdown line *vs.* non-transformant under 7-d Fe deficiency (Ogo et al. [2008]); *IRO2 6d*, roots from an *IRO2* knockdown line *vs.* non-transformant under 6-day Fe deficiency (Ogo et al. [2007]); *HRZ1/2 Cont. and 7d*, roots from *OsHRZ1* and *OsHRZ2* knockdown lines *vs.* non-transformant under Fe-sufficient control condition and 7-day Fe deficiency treatment, respectively (Kobayashi et al. [2013]).

^a^OsYSL16 is also proposed as a copper-NA transporter involved in internal copper distribution (Zheng et al. [2012]).

^b^The probe used for Northern blotting analysis (Kobayashi et al. [2005]) may not have differentiated between *OsMTK1* and *OsMTK2*.

^c^Downregulation was observed in the *IDEF1* induction lines, but upregulation was not observed in the *IDEF1* knockdown lines, suggesting that negative regulation by IDEF1 may be a secondary effect (Kobayashi et al. [2009]).

^d^Downregulation was observed in both the *IDEF1* induction and knockdown lines. Positive regulation by IDEF1 may be more plausible, because downregulation in the knockdown lines was more dominant and may reflect more direct effects than overexpression lines.

^e^Confirmation by quantitative RT-PCR has been conducted using plants grown on calcareous soil (Ogo et al. [2011]), but not with hydroponically grown plants.

In barley, the secretion of MAs follows a diurnal pattern, with a sharp peak in the morning (Takagi et al. [1984]). MAs are thought to be synthesized in vesicles that can be observed within root cells; these vesicles are swollen in the early morning and shrink by the evening. These vesicles may therefore be putative sites of MAs biosynthesis and storage until secretion in the morning, and thus are designated MAs-vesicles (Nishizawa and Mori [1987]; Negishi et al. [2002]). Rice also shows diurnal fluctuations in MAs secretion and the presence of similar vesicles, although they are much less obvious than in barley roots (Nozoye et al. [2014]), presumably due to the much lower amounts of MAs synthesized in rice (Mori et al. [1991]; Kanazawa et al. [1994]). In Fe-deficient rice root cells, tagging with green fluorescent protein (GFP) revealed that OsNAS2 is localized to small vesicles, presumably the MAs-vesicles, and that these vesicles move dynamically within the cell (Nozoye et al. [2014]). Mutation of either the tyrosine or di-leucine motif conserved in all known NASs disrupts OsNAS2-GFP vesicle formation and movement, and abolishes normal NAS function (Nozoye et al. [2014]). The MAs are thought to be transported out of the MAs-vesicle to the cytosol near the periphery of root cells before subsequent secretion to the rhizosphere. In rice, TOM1 transporter mediates the DMA secretion through the plasma membrane (Nozoye et al. [2011]). Transcript level of *TOM1*, as well as genes involved in DMA biosynthesis such as *OsNAS1*, *OsNAS2*, and *OsNAAT1*, shows daily fluctuations (Nozoye et al. [2004], [2011]). This regulation, in addition to the formation and trafficking of MAs-vesicles, is thought to contribute to the observed pattern of DMA secretion in the morning.

The Fe(III)-DMA complex formed in the rhizosphere is then taken up into root cells via the OsYSL15 transporter (Figure 1A) (Inoue et al. [2009]; Lee et al. [2009]). Transcript abundance of *OsYSL15* also shows daily fluctuation (Inoue et al. [2009]), possibly supporting efficient Fe uptake. Moreover, expression of all the above-mentioned enzymes and transporters for DMA-based Fe uptake is strongly up-regulated under conditions of Fe deficiency to meet the increased demand of Fe uptake (Table 1). OsYSL16 is another Fe(III)-DMA transporter that is expressed in the plasma membrane of root epidermis/exodermis (Kakei et al. [2012]; Lee et al. [2012]), and therefore may also mediate Fe(III)-DMA uptake from the rhizosphere. However, in contrast to the strong induction of *OsYSL15*, expression of the *OsYSL16* gene is constitutive and only slightly induced under conditions of Fe deficiency, suggesting that Fe(III)-DMA uptake is predominantly mediated through OsYSL15.

In addition to Fe(III)-DMA uptake, rice also possesses the components of an Fe^2+^ uptake system (Figure 1B) (Ishimaru et al. [2006]). The epidermis and exodermis of rice roots express various Fe^2+^ transporters in the plasma membrane, including OsIRT1, OsIRT2, OsNRAMP1, and OsNRAMP5 (Ishimaru et al. [2006], [2012]; Takahashi et al. [2011]; Ogo et al. [2014]). Among these, OsIRT1 is thought to be the primary transporter involved in Fe^2+^ uptake (Ishimaru et al. [2006]). The transcript levels of the *OsIRT1*, *OsIRT2*, and *OsNRAMP1*, but not *OsNRAMP5*, are strongly upregulated under conditions of Fe deficiency (Table 1). OsNRAMP5 mediates the predominant pathway for manganese and cadmium uptake, but has relatively small contribution to Fe uptake under Fe-deficient conditions (Ishikawa et al. [2012]; Ishimaru et al. [2012]; Sasaki et al. [2012]).

In the Strategy I system utilized by non-graminaceous plants, Fe^2+^ uptake is coupled to ferric-chelate reductase activity on the root surface, which is strongly induced under conditions of Fe deficiency (Römheld and Marschner [1986]). However, rice roots show very low ferric-chelate reductase activity with no induction response to Fe deficiency (Ishimaru et al. [2006]), thus it lacks complete Strategy I system. This is likely because paddy rice has adapted to anaerobic conditions in which Fe^2+^ is abundant, making direct uptake without active ferric-chelate reduction sufficient for Fe acquisition.

Strategy I plants secrete protons and various phenolic compounds into the rhizosphere under conditions of low Fe availability; this serves to increase Fe solubility and maintain ferric-chelate reductase activity (Römheld and Marschner [1986]; Rodríguez-Celma et al. [2013]; Fourcroy et al. [2014]; Schmid et al. [2014]). Rice also possesses phenolics efflux transporters (PEZ1 and PEZ2) (Ishimaru et al. [2011]; Bashir et al. [2011a]), among which PEZ2 is expressed in the plasma membrane of root epidermis/exodermis and is thought to be responsible for the secretion of protocatechuic acid and caffeic acid into the rhizosphere (Bashir et al. [2011a]; Ogo et al. [2014]). These phenolic compounds possess chemical properties to chelate and reduce Fe in vitro (Yoshino and Murakami [1998]), which may contribute to Fe^2+^ uptake, although this effect may be limited as the *PEZ2* transcript shows little induction under conditions of Fe deficiency (Table 1).

### Translocation of iron to the shoots

**Figure 2:**
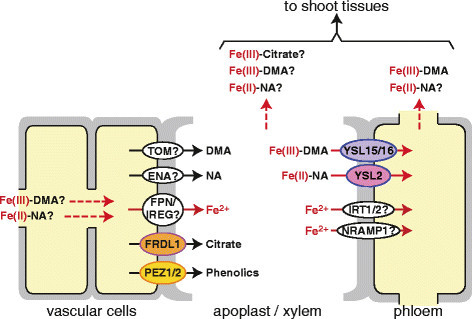
**Fe translocation in vascular cells of rice roots.** Molecules involved in xylem and phloem loading of Fe. Ovals represent transporters. Putative involvement of transporters and Fe-chelates in Fe translocation is indicated by question marks. Red arrows with broken lines indicate translocation of Fe-chelates.

Following uptake from the rhizosphere into the root epidermis/exodermis, Fe is transported toward the vascular bundle for translocation to the shoots via xylem and phloem. This radial transport system occurs through both symplasmic and apoplasmic pathways, but the latter pathway is impeded by two Casparian strips in the exodermis and endodermis (Enstone et al. [2002]). To avoid Fe toxicity and facilitate its transport, the greater portions of both ferric and ferrous cellular Fe are chelated. In rice, DMA, nicotianamine (NA), and citric acid are thought to be the dominant Fe chelators. Figure 2 depicts the Fe translocation in vascular cells and possible involvement of Fe chelators and transporters.

Under Fe-deficient conditions, the enzymes and transporters responsible for Fe uptake are induced not only in the epidermis/exodermis, but also in the cortex and vascular bundle (Table 1) (Inoue et al. [2003], [2008], [2009]; Bashir et al. [2006]; Ishimaru et al. [2006]; Lee et al. [2009]; Nozoye et al. [2011]; Ogo et al. [2014]), where they are thought to be involved in Fe transport to shoot tissues. DMA has been detected in rice xylem and phloem sap (Mori et al. [1991]; Kakei et al. [2009]). Moreover, the Fe(III)-DMA complex has been identified as the primary chemical form of Fe in phloem sap (Nishiyama et al. [2012]). These findings indicate that DMA is responsible not only for Fe uptake from the rhizosphere, but also for internal Fe translocation. OsYSL15 and OsYSL16 are expressed in vascular bundles, where they are thought to transport Fe(III)-DMA for phloem Fe transport (Inoue et al. [2009]; Lee et al. [2009], [2012]; Kakei et al. [2012]).

NA is a precursor of DMA, and is biosynthesized by the NAS enzyme in all plant species analyzed to date, including non-graminaceous species (Shojima et al. [1989]; reviewed by Hell and Stephan [2003]; Curie et al. [2009]). NA functions as a potent chelator of Fe and other divalent metals, facilitating their translocation within the plant while suppressing their toxicity. In rice, NA is biosynthesized by three NAS enzymes (OsNAS1, OsNAS2, and OsNAS3). Both *OsNAS1* and *OsNAS2* expression are induced in whole roots in response to Fe deficiency, whereas *OsNAS3* expression is weaker and detected mainly in the vascular bundle, suggesting a role in Fe translocation (Inoue et al. [2003]). The OsYSL2 transporter is responsible for Fe(II)-NA and manganese(II)-NA transport across the plasma membrane, and plays a crucial role in phloem-mediated Fe distribution (Koike et al. [2004]; Ishimaru et al. [2010]). The NA efflux transporters ENA1 and ENA2 (Nozoye et al. [2011]) are thought to be responsible for NA extrusion to the apoplast or intracellular compartments for redistribution of Fe. The expression of *OsYSL2* and *ENA1* are strongly induced under conditions of Fe deficiency (Table 1) (Koike et al. [2004]; Lee et al. [2009]; Ishimaru et al. [2010]; Nozoye et al. [2011]; Ogo et al. [2014]).

Citrate is an Fe(III) chelator and is thought to play a dominant role in xylem Fe transport in non-graminaceous plants (Rellán-Álvarez et al. [2010]). In rice, the OsFRDL1 transporter mediates citrate efflux into the xylem for efficient Fe translocation (Yokosho et al. [2009]). *OsFRDL1* is specifically expressed in root pericycle cells, with no apparent induction under conditions of Fe deficiency (Table 1) (Inoue et al. [2004]; Yokosho et al. [2009]; Ogo et al. [2014]).

The recent identification of the plasma membrane-localized protocatechuic acid transporters PEZ1 and PEZ2 indicated that phenolics are also involved in Fe utilization within the rice plant (Ishimaru et al. [2011]; Bashir et al. [2011a]). *PEZ1* is expressed specifically in the vascular bundle, while *PEZ2* is expressed in the epidermis/exodermis, cortex, and vascular bundle (Ishimaru et al. [2011]; Bashir et al. [2011a]; Ogo et al. [2014]). Both *PEZ1* and *PEZ2* expression are only moderately induced under conditions of Fe deficiency in vascular bundles. Knockdown or knockout mutants of either *PEZ1* or *PEZ2* expression show decreased amounts of protocatechuic acid and caffeic acid, as well as decreased Fe concentrations, in xylem sap.

As xylem is an apoplasmic space, Fe must be effluxed out of the cell for xylem loading, after passing through the Casparian strip at the endodermis. The transporter responsible for this Fe efflux has yet to be identified in plants, although the ferroportin 1/iron-regulated 1 protein identified in *Arabidopsis thaliana* (AtFPN1/AtIREG1) is a likely candidate (Morrissey et al. [2009]) for the Fe^2+^ efflux transporter. The effluxed Fe would be rapidly chelated either as ferrous form or ferric form after chemical or enzymatic oxidization. On the other hand, Fe^2+^ transport into the cytosol might occur via OsIRT1, OsIRT2, or OsNRAMP1, because expression of these transporters are induced under Fe deficiency in the vascular bundle in addition to the epidermis/exodermis and cortex (Table 1) (Ishimaru et al. [2006]; Ogo et al. [2014]).

The subcellular transport of Fe is crucial for cellular function, and can also affect Fe translocation. As vacuoles constitute a large proportion of the total cellular space, vacuolar Fe transport is thought to substantially affect Fe flux. OsVIT1 and OsVIT2 are thought to transport Fe across the tonoplast into the vacuole in rice (Zhang et al. [2012]). The mitochondrial iron transporter (MIT) transports Fe into mitochondria, and disruption of this gene is lethal (Bashir et al. [2011b]). Although *OsVIT1*, *OsVIT2*, and *MIT* expression are repressed under conditions of Fe deficiency (Table 1) (Bashir et al. [2011b]; Zhang et al. [2012]), the contribution of these transporters to root Fe flux is not well understood.

### Regulation of root iron responses

**Figure 3:**
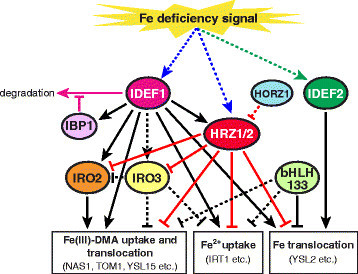
**Regulation of Fe deficiency responses in rice roots.** Ovals indicate regulatory proteins. Boxes indicate proteins responsible for Fe uptake and translocation. All depicted proteins except IDEF1, IDEF2, and OsHORZ1 are transcriptionally induced in response to Fe deficiency. Broken lines indicate putative pathways. Line colors indicate the type of regulation: black lines, transcriptional regulation; pink lines, IDEF1 protein degradation and its inhibition; red lines, unknown mechanism of regulation occurring primarily under Fe sufficiency, which may involve protein ubiquitination by OsHRZs; blue lines, putative Fe sensing by IDEF1 and OsHRZs via direct binding of Fe and other metals; green line, putative Fe sensing by IDEF2 through an unknown mechanism.

As reviewed above, the levels of expression of numerous rice genes involved in Fe uptake and translocation are strongly induced under conditions of Fe deficiency at the transcriptional level. Highly conserved temporal and spatial patterns of expression are observed, especially with regard to genes involved in DMA-based Fe uptake (Kobayashi et al. [2005]; Itai et al. [2013]; Ogo et al. [2014]). These Fe deficiency responses are mediated by several regulators, which are summarized in Figure 3.

IDEF1 and IDEF2 are transcription factors that specifically bind the Fe deficiency-responsive *cis*-acting elements IDE1 and IDE2, respectively (Kobayashi et al. [2007]; Ogo et al. [2008]). IDE1 and IDE2 have been identified from the barley Fe deficiency-inducible gene *IDS2*, but they are also able to function in various other species, including rice and non-graminaceous plants (Kobayashi et al. [2003], [2004]). IDEF1 and IDEF2 positively regulate the expression of subsets of Fe deficiency-induced genes with relatively little overlap (Kobayashi et al. [2007], [2009]; Ogo et al. [2008]). IDEF1 regulates most genes known to be involved in Fe(III)-DMA and Fe^2+^ uptake, as well as Fe translocation, especially during the early stages of Fe deficiency. IDEF2 regulates *OsYSL2* and other Fe deficiency-inducible genes which might be involved in Fe translocation (Table 1, Figure 3). At later stages of Fe deficiency, IDEF1 shifts its pattern of regulation, with decreased impact on Fe uptake-related genes (Table 1) (Kobayashi et al. [2009]). Analysis of transgenic rice plants with induced or repressed expression of *IDEF1* revealed a positive correlation between *IDEF1* expression level and Fe deficiency tolerance during the early stages of Fe deficiency, but not after prolonged deficiency (Kobayashi et al. [2007], [2009], [2013]). *IDEF2* knockdown results in aberrant Fe distribution between roots and shoots (Ogo et al. [2008]), whereas *IDEF1* disruption does not appear to affect Fe distribution among plant organs (Kobayashi et al. [2007], [2013]). Notably, neither *IDEF1* nor *IDEF2* is induced by Fe deficiency (Kobayashi et al. [2007], [2009], [2010]; Ogo et al. [2008]; Itai et al. [2013]), suggesting their potential roles in sensing upstream signaling in the Fe deficiency response cascade (Figure 3). Two homologous Bowman–Birk trypsin inhibitors, designated IBP1.1 and IBP1.2, were found to interact with IDEF1 in yeast two-hybrid assay (Zhang et al. [2014]). Both *IBP1.1* and *IBP1.2* expression are induced by Fe deficiency and positively regulated by IDEF1 (Table 1) (Zhang et al. [2014]). Using a transient expression system in rice protoplast and an in vitro experiment, IBP1.1 was found to protect IDEF1 protein from degradation which was deduced to be mediated via the 26S proteasome pathway (Zhang et al. [2014]). These results suggest that IBP1.1 fine tunes IDEF1 protein expression for optimal function during Fe deficiency (Figure 3). Although it is not known whether IBP1.2 also inhibits IDEF1 degradation, IBP1.2 might also regulate IDEF1 function redundantly. Alternatively, IBP1.1 and IBP1.2 might participate in regulating IDEF1 function in different stages of Fe deficiency, because the Fe deficiency-induced expression of *IBP1.1* and *IBP1.2* shows different time-course properties; *IBP1.1* expression is higher at day 1 of Fe deficiency compared with day 7, whereas *IBP1.2* expression is higher at day 7 of Fe deficiency (Zhang et al. [2014]).

Three basic helix-loop-helix transcription factors, OsIRO2, OsIRO3 and OsbHLH133, are involved in Fe deficiency response in rice (Figure 3) (Ogo et al. [2006], [2007], [2011]; Zheng et al. [2010]; Wang et al. [2013]). These factors are transcriptionally induced under conditions of Fe deficiency; they have been found and characterized from the Fe deficiency-induced genes in microarray analyses (Ogo et al. [2006]; Zheng et al. [2010]; Wang et al. [2013]). OsIRO2 is a positive regulator of most genes known to be involved in Fe(III)-DMA uptake and translocation (Table 1, Figure 3) (Ogo et al. [2007], [2011]). Overexpression of OsIRO2 enhances DMA secretion (Ogo et al. [2007]) and confers substantial tolerance to low Fe availability in calcareous soil, significantly improving both plant biomass and seed yield (Ogo et al. [2011]). Expression of *OsIRO2* itself is positively regulated by IDEF1, forming a transcriptional cascade enhancing the expression of genes involved in Fe(III)-DMA uptake and translocation (Figure 3) (Kobayashi et al. [2007], [2009]). In contrast, OsIRO3 and OsbHLH133 are thought to negatively regulate Fe deficiency responses (Zheng et al. [2010]; Wang et al. [2013]). OsIRO3 overexpression lines suggest that this factor negatively regulates the Fe deficiency-inducible genes *OsIRO2*, *OsNAS1*, *OsNAS2*, *OsYSL15*, *OsIRT1*, and *OsNRAMP1* (Zheng et al. [2010]). However, *OsIRO3* knockdown or knockout studies required to clarify the precise function of this protein have yet to be reported. Microarray results suggested that *OsIRO3* expression may be positively regulated by IDEF1 (Table 1). OsbHLH133 negatively regulates Fe translocation from root to shoot (Wang et al. [2013]). *OsbHLH133* knockout mutants show slightly higher expression of some Fe deficiency-inducible genes, including *OsNAS1*, *OsNAS2*, *TOM1*, *OsYSL15*, *OsNRAMP1*, and *OsYSL2*, mainly under Fe-sufficient conditions (Wang et al. [2013]).

DNA sequences specifically recognized by IDEF1, IDEF2, and OsIRO2 have been identified biochemically (Kobayashi et al. [2007]; Ogo et al. [2006], [2008]). Recent *in silico* prediction of *cis*-sequences over-represented in Fe deficiency-induced gene promoters revealed that the IDEF1-, IDEF2-, and OsIRO2-binding sequences are enriched within the 500-bp promoter regions of Fe deficiency-induced genes (Kakei et al. [2013]; Ogo et al. [2014]). The enrichment of IDEF1-binding sequences (the core sequence of which is CATGC) is particularly remarkable, strongly suggesting the importance of IDEF1 in the Fe deficiency response. The simulation also predicted enrichment of novel candidate *cis*-sequences, such as ACGTACGT (designated FAM1 for Fe deficiency-associated motif 1) and AGCTAGCT (designated DCEp1 for putative downstream core element 1), as well as common regulatory sequences, such as TATA-box and upstream TFIIB-recognition elements near the transcription start sites of Fe deficiency-inducible genes (Kakei et al. [2013]). Regulatory proteins interacting with these sequences may play important roles in Fe deficiency responses.

IDEF1 binds ferrous Fe and other divalent metals, such as zinc (Zn) and nichel, via its histidine-asparagine repeats and proline-rich regions (Kobayashi et al. [2012]). In rice, deletion of these metal-binding regions abolishes the ability of overexpressed IDEF1 to transactivate downstream genes during the early stages of Fe deficiency (Kobayashi et al. [2012]). These results suggest that IDEF1 may function as a sensor of Fe deficiency, in which the signal detection may be determined by the relative concentrations of Fe *vs.* other metals.

Recently, another kind of Fe-binding regulators, OsHRZ1 and OsHRZ2, have been identified (Kobayashi et al. [2013]) from the Fe deficiency-induced genes listed in previous microarray analysis (Ogo et al. [2006]). OsHRZ1 and OsHRZ2 share hemerythrin domains that bind Fe and Zn (Kobayashi et al. [2013]), suggesting that they are candidate Fe sensors. OsHRZ1 and OsHRZ2 also possess protein ubiquitination activity presumably mediated by RING Zn-finger domains, which are deduced to be involved in protein degradation or modification, even though the target proteins for ubiquitination have not been clarified (Kobayashi et al. [2013]). In addition, OsHRZ1 and OsHRZ2 also possess CHY and CTCHY Zn-finger domains that may mediate transcriptional or post-transcriptional gene regulation. Transgenic rice lines subjected to *OsHRZ1* and *OsHRZ2* knockdown show substantial tolerance to low Fe availability, and accumulate large amounts of Fe in shoots and seeds irrespective of Fe nutritional conditions. These knockdown lines also show enhanced expression of Fe deficiency-inducible genes involved in Fe uptake and/or utilization even under Fe-sufficient conditions, but this enhancement is much less obvious under prolonged Fe deficiency (Table 1) (Kobayashi et al. [2013]). These results indicate that OsHRZ1 and OsHRZ2 are negative regulators of various Fe deficiency-inducible genes mostly under Fe sufficiency (Figure 3). OsHRZ1 and OsHRZ2 may sense Fe signals via direct binding of Fe and Zn, and prevent excess Fe uptake under conditions of Fe sufficiency. Both *OsHRZ1* and *OsHRZ2* expression are induced under conditions of Fe deficiency and are positively regulated by IDEF1, forming a negative feedback loop of the IDEF1 pathway (Figure 3). In *Arabidopsis*, the OsHRZ homolog BRUTUS (BTS) interacts indirectly with the OsIRO3 homolog POPEYE (PYE) by binding another subset of basic helix-loop-helix transcription factors (Long et al. [2010]). *BTS* and *PYE* have been identified from Fe deficiency-induced genes in pericycle of *Arabidopsis* roots (Long et al. [2010]). *BTS* knockdown show enhanced tolerance to Fe deficiency, whereas *PYE* knockout results in susceptibility to Fe deficiency. PYE negatively regulates a subset of Fe translocation-related genes. Whether BTS is involved in this pathway has not been clarified (Long et al. [2010]).

In addition to OsHRZ1 and OsHRZ2, rice possesses another protein carrying a hemerythrin domain, designated OsHORZ1, which lacks other domains conserved in OsHRZs (Kobayashi et al. [2013]). *OsHORZ1* knockdown show increased susceptibility to Fe deficiency and repression of Fe deficiency-inducible genes (Kobayashi et al. [2013]). Thus, OsHORZ1 appears to possess a function opposite to OsHRZs. This function may be attributed to domain characteristics of OsHORZ1 that may antagonize OsHRZ function (Figure 3). *OsHORZ1* expression shows only slight induction in response to Fe deficiency (Table 1) (Kobayashi et al. [2013]).

In addition to specific regulatory interactions, the expression of Fe deficiency-inducible genes is also affected by various plant hormones, such as ethylene, auxin, gibberellin, jasmonic acid and abscisic acid. Ethylene precursor treatment enhances the expression of *OsNAS1*, *OsNAS2*, *OsYSL15*, and *OsIRT1* under both Fe sufficiency and deficiency, and this enhancement may be mediated by the OsIRO2 pathway (Wu et al. [2011]). Knockout of a transcription factor involved in auxin response, *OsARF12*, also affects expression of some Fe deficiency-induced genes including *OsIRT1* in roots (Qi et al. [2011]). Gibberellin and jasmonic acid induce *OsIRO2* and *IBP1* expression, respectively (Komatsu and Takasaki [2009]; Yoshii et al. [2010]). A jasmonic acid-induced receptor-like protein (OsRMC) that negatively regulates jasmonic acid-mediated root development (Jiang et al. [2007]) has recently been identified as a positive regulator of the Fe deficiency-inducible genes *OsNAS1*, *OsNAS2*, *OsNAAT1*, *OsDMAS1*, *OsYSL15*, *OsIRT1*, and *OsIRO2* (Yang et al. [2013]). *OsRMC* expression is transcriptionally induced by jasmonic acid and during the early stages of Fe deficiency, but is repressed under prolonged Fe deficiency (Table 1) (Jiang et al. [2007]; Yang et al. [2013]). On the other hand, some IDEF1-induced genes activated in later stages of Fe deficiency are also regulated by abscisic acid (Kobayashi et al. [2009]). In fact, IDEF1 belongs to the ABI3 (B3) transcription factor family, involved in mediating the abscisic acid response (Kobayashi et al. [2007]).

In Strategy-I plants, systemic Fe signal derived from shoots is thought to determine whether Fe deficiency response in roots takes place (Giehl et al. [2009]; García et al. [2013]). This signal is proposed to be transmitted from shoot to root via phloem, locating upstream of the induction system of Fe deficiency-responsive gene expression mediated by auxin and ethylene (García et al. [2013]). Mechanism of this systemic regulation is less understood in rice, although the existence of similar regulation has been suggested (Enomoto et al. [2009]). Thus, the Fe deficiency response is a complicated process that reflects various input signals to maintain adequate amounts of available Fe for cellular activities.

## Conclusions

Rice responds to low Fe availability by inducing enzymes, transporters, and regulators that participate in Fe uptake from the rhizosphere and Fe translocation within the plant body. Recent studies demonstrated that rice utilizes both classical DMA-based Fe uptake and direct Fe^2+^ uptake. Research has also elucidated the function of DMA in internal Fe translocation, in addition to its known role in Fe uptake. The differential spatial expression of genes responsible for Fe uptake and translocation in response to Fe deficiency is highly coordinated and is well correlated with their physiological functions. In addition to positive regulation of these genes at the transcriptional level, negative regulation at both the transcriptional and translational levels may also function in fine tuning the Fe deficiency response. Although the Fe-sensing mechanism remains unclear, further characterization of IDEF1 and OsHRZs may in fact consolidate their respective roles as Fe sensors. Despite these advances in our understanding, a potentially large proportion of the molecules involved in intercellular and subcellular Fe movement have not yet been identified. Further investigation into these issues will help to develop novel tools for producing Fe-efficient and Fe-fortified crops with increased versatility.

## Authors' contributions

TK and RNI analyzed the microarray data. TK wrote the manuscript with critical revision by RNI and NKN. All of the authors read and approved the manuscript.

## Authors’ original submitted files for images

Below are the links to the authors’ original submitted files for images. Authors’ original file for figure 1 Authors’ original file for figure 2 Authors’ original file for figure 3

## References

[CR1] Bashir K, Inoue H, Nagasaka S, Takahashi M, Nakanishi H, Mori S, Nishizawa NK (2006). Cloning and characterization of deoxymugineic acid synthase genes from graminaceous plants. J Biol Chem.

[CR2] Bashir K, Ishimaru Y, Shimo H, Kakei Y, Senoura T, Takahashi R, Sato Y, Sato Y, Uozumie N, Nakanishi H, Nishizawa NK (2011). Rice phenolics efflux transporter 2 (PEZ2) plays an important role in solubilizing apoplasmic iron. Soil Sci Plant Nutr.

[CR3] Bashir K, Ishimaru Y, Shimo H, Nagasaka S, Fujimoto M, Takanashi H, Tsutsumi N, An G, Nakanishi H, Nishizawa NK (2011). The rice mitochondrial iron transporter is essential for plant growth. Nat Commun.

[CR4] Bashir K, Nozoye T, Ishimaru Y, Nakanishi H, Nishizawa NK (2013). Exploiting new tools for iron bio-fortification of rice. Biotechnol Adv.

[CR5] Cheng L, Wang F, Shou H, Huang F, Zheng L, He F, Li J, Zhao FJ, Ueno D, Ma JF, Wu P (2007). Mutation in nicotianamine aminotransferase stimulated the Fe(II) acquisition system and led to iron accumulation in rice. Plant Physiol.

[CR6] Curie C, Cassin G, Couch D, Divol F, Higuchi K, Le Jean M, Misson J, Schikora A, Czernic P, Mari S (2009). Metal movement within the plant: contribution of nicotianamine and yellow stripe 1-like transporters. Annals Bot.

[CR7] Enomoto Y, Hashida S, Shoji K, Shimada H, Yoshihara T, Goto F (2009). Expressions of iron uptake genes in roots are affected by long-distance signals both in non-graminaceous and in graminaceous plants. Proceedings of XVI International Plant Nutrition Colloquium.

[CR8] Enstone DE, Peterson CA, Ma F (2002). Root endodermis and exodermis: structure, function, and responses to the environment. J Plant Growth Regul.

[CR9] Fourcroy P, Sisó-Terraza P, Sudre D, Sabirón M, Reyt G, Gaymard F, Abadía A, Abadía J, Álvarez-Fernández A, Briat JF (2014). Involvement of the ABCG37 transporter in secretion of scopoletin and derivatives by Arabidopsis roots in response to iron deficiency. New Phytol.

[CR10] García MJ, Romera FJ, Stacey MG, Stacey G, Villar E, Alcántara E, Pérez-Vicente R (2013). Shoot to root communication is necessary to control the expression of iron-acquisition genes in Strategy I plants. Planta.

[CR11] Giehl RFH, Meda AR, von Wirén N (2009). Moving up, down, and everywhere: signaling of micronutrients in plants. Curr Opin Plant Biol.

[CR12] Hell R, Stephan UW (2003). Iron uptake, trafficking and homeostasis in plants. Planta.

[CR13] Higuchi K, Suzuki K, Nakanishi H, Yamaguchi H, Nishizawa NK, Mori S (1999). Cloning of nicotianamine synthase genes, novel genes involved in the biosynthesis of phytosiderophores. Plant Physiol.

[CR14] Higuchi K, Watanabe S, Takahashi M, Kawasaki S, Nakanishi H, Nishizawa NK, Mori S (2001). Nicotianamine synthase gene expression differs in barley and rice under Fe-deficient conditions. Plant J.

[CR15] Inoue H, Higuchi K, Takahashi M, Nakanishi H, Mori S, Nishizawa NK (2003). Three rice nicotianamine synthase genes, *OsNAS1*, *OsNAS2*, and *OsNAS3* are expressed in cells involved in long-distance transport of iron and differentially regulated by iron. Plant J.

[CR16] Inoue H, Suzuki M, Takahashi M, Nakanishi H, Mori S, Nishizawa NK (2004). A rice FRD3-like (OsFRDL1) gene is expressed in the cells involved in long-distance transport. Soil Sci Plant Nutr.

[CR17] Inoue H, Takahashi M, Kobayashi T, Suzuki M, Nakanishi H, Mori S, Nishizawa NK (2008). Identification and localisation of the rice nicotianamine aminotransferase gene *OsNAAT1* expression suggests the site of phytosiderophore synthesis in rice. Plant Mol Biol.

[CR18] Inoue H, Kobayashi T, Nozoye T, Takahashi M, Kakei Y, Suzuki K, Nakazono M, Nakanishi H, Mori S, Nishizawa NK (2009). Rice OsYSL15 is an iron-regulated iron(III)-deoxymugineic acid transporter expressed in the roots and is essential for iron uptake in early growth of the seedlings. J Biol Chem.

[CR19] Ishikawa S, Ishimaru Y, Igura M, Kuramata M, Abe T, Senoura T, Hase Y, Arao T, Nishizawa NK, Nakanishi H (2012). Ion-beam irradiation, gene identification, and marker-assisted breeding in the development of low-cadmium rice. Proc Natl Acad Sci U S A.

[CR20] Ishimaru Y, Suzuki M, Tsukamoto T, Suzuki K, Nakazono M, Kobayashi T, Wada Y, Watanabe S, Matsuhashi S, Takahashi M, Nakanishi H, Mori S, Nishizawa NK (2006). Rice plants take up iron as an Fe^3+^-phytosiderophore and as Fe^2+^. Plant J.

[CR21] Ishimaru Y, Masuda H, Bashir K, Inoue H, Tsukamoto T, Takahashi M, Nakanishi H, Aoki N, Hirose T, Ohsugi R, Nishizawa NK (2010). Rice metal-nicotianamine transporter, OsYSL2, is required for the long-distance transport of iron and manganese. Plant J.

[CR22] Ishimaru Y, Kakei Y, Shimo H, Bashir K, Sato Y, Sato Y, Uozumi N, Nakanishi H, Nishizawa NK (2011). A rice phenolic efflux transporter is essential for solubilizing precipitated apoplasmic iron in the plant stele. J Biol Chem.

[CR23] Ishimaru Y, Takahashi R, Bashir K, Shimo H, Senoura T, Sugimoto K, Ono K, Yano M, Ishikawa S, Arao T, Nakanishi H, Nishizawa NK (2012). Characterizing the role of rice NRAMP5 in manganese, iron and cadmium transport. Sci Rep.

[CR24] Itai RN, Ogo Y, Kobayashi T, Nakanishi H, Nishizawa NK (2013). Rice genes involved in phytosiderophore biosynthesis are synchronously regulated during the early stages of iron deficiency in roots. Rice.

[CR25] Jiang J, Li J, Xu Y, Han Y, Bai Y, Zhou G, Lou Y, Xu Z, Chong K (2007). RNAi knockdown of *Oryza sativa root meander curling* gene led to altered root development and coiling which were mediated by jasmonic acid signaling in rice. Plant Cell Environ.

[CR26] Kakei Y, Yamaguchi I, Kobayashi T, Takahashi M, Nakanishi H, Yamakawa T, Nishizawa NK (2009). A highly sensitive, quick, and simple quantification method for nicotianamine and 2′-deoxymugineic acid from minimum samples using LC/ESI-TOF-MS achieves functional analysis of these components in plants. Plant Cell Physiol.

[CR27] Kakei Y, Ishimaru Y, Kobayashi T, Yamakawa T, Nakanishi H, Nishizawa NK (2012). OsYSL16 plays a role in the allocation of iron. Plant Mol Biol.

[CR28] Kakei Y, Ogo Y, Itai RN, Kobayashi T, Yamakawa T, Nakanishi H, Nishizawa NK (2013). Development of a novel prediction method of *cis*-elements to hypothesize collaborative functions of *cis*-element pairs in iron-deficient rice. Rice.

[CR29] Kanazawa K, Higuchi K, Nishizawa NK, Fushiya S, Chino M, Mori S (1994). Nicotianamine aminotransferase activities are correlated to the phytosiderophore secretions under Fe-deficient conditions in Gramineae. J Exp Bot.

[CR30] Kawai S, Takagi S, Sato Y (1988). Mugineic acid-family phytosiderophores in root-secretions of barley, corn and sorghum varieties. J Plant Nutr.

[CR31] Kobayashi T, Nishizawa NK (2012). Iron uptake, translocation, and regulation in higher plants. Annu Rev Plant Biol.

[CR32] Kobayashi T, Nakayama Y, Itai RN, Nakanishi H, Yoshihara T, Mori S, Nishizawa NK (2003). Identification of novel *cis*-acting elements, IDE1 and IDE2, of the barley *IDS2* gene promoter conferring iron-deficiency-inducible, root-specific expression in heterogeneous tobacco plants. Plant J.

[CR33] Kobayashi T, Nakayama Y, Takahashi M, Inoue H, Nakanishi H, Yoshihara T, Mori S, Nishizawa NK (2004). Construction of artificial promoters highly responsive to iron deficiency. Soil Sci Plant Nutr.

[CR34] Kobayashi T, Suzuki M, Inoue H, Itai RN, Takahashi M, Nakanishi H, Mori S, Nishizawa NK (2005). Expression of iron-acquisition-related genes in iron-deficient rice is co-ordinately induced by partially conserved iron-deficiency-responsive elements. J Exp Bot.

[CR35] Kobayashi T, Ogo Y, Itai RN, Nakanishi H, Takahashi M, Mori S, Nishizawa NK (2007). The transcription factor IDEF1 regulates the response to and tolerance of iron deficiency in plants. Proc Natl Acad Sci U S A.

[CR36] Kobayashi T, Itai RN, Ogo Y, Kakei Y, Nakanishi H, Takahashi M, Nishizawa NK (2009). The rice transcription factor IDEF1 is essential for the early response to iron deficiency, and induces vegetative expression of late embryogenesis abundant genes. Plant J.

[CR37] Kobayashi T, Ogo Y, Aung MS, Nozoye T, Itai RN, Nakanishi H, Yamakawa T, Nishizawa NK (2010). The spatial expression and regulation of transcription factors IDEF1 and IDEF2. Annals Bot.

[CR38] Kobayashi T, Itai RN, Aung MS, Senoura T, Nakanishi H, Nishizawa NK (2012). The rice transcription factor IDEF1 directly binds to iron and other divalent metals for sensing cellular iron status. Plant J.

[CR39] Kobayashi T, Nagasaka S, Senoura T, Itai RN, Nakanishi H, Nishizawa NK (2013). Iron-binding haemerythrin RING ubiquitin ligases regulate plant iron responses and accumulation. Nat Commun.

[CR40] Koike S, Inoue H, Mizuno D, Takahashi M, Nakanishi H, Mori S, Nishizawa NK (2004). *OsYSL2* is a rice metal-nicotianamine transporter that is regulated by iron and expressed in the phloem. Plant J.

[CR41] Komatsu S, Takasaki H (2009). Gibberellin-regulated gene in the basal region of rice leaf sheath encodes basic helix–loop–helix transcription factor. Amino Acids.

[CR42] Lee S, Chiecko JC, Kim SA, Walker EL, Lee Y, Guerinot ML, An G (2009). Disruption of *OsYSL15* leads to iron inefficiency in rice plants. Plant Physiol.

[CR43] Lee S, Ryoo N, Jeon JS, Guerinot ML, An G (2012). Activation of rice *Yellow Stripe1-Like 16* (*OsYSL16*) enhances iron efficiency. Mol Cell.

[CR44] Long TA, Tsukagoshi H, Busch W, Lahner B, Salt D, Benfey PN (2010). The bHLH transcription factor POPEYE regulates response to iron deficiency in *Arabidopsis* roots. Plant Cell.

[CR45] Marschner H (1995). Mineral Nutrition of Higher Plants.

[CR46] Masuda H, Aung MS, Nishizawa NK (2013). Iron biofortification of rice using different transgenic approaches. Rice.

[CR47] Mori S, Nishizawa N (1987). Methionine as a dominant precursor of phytosiderophores in *Graminaceae* plants. Plant Cell Physiol.

[CR48] Mori S, Nishizawa N, Hayashi H, Chino M, Yoshimura E, Ishihara J (1991). Why are young rice plants highly susceptible to iron deficiency?. Plant Soil.

[CR49] Morrissey J, Baxter IR, Lee J, Li L, Lahner B, Grotz N, Kaplan J, Salt DE, Guerinot ML (2009). The ferroportin metal efflux proteins function in iron and cobalt homeostasis in *Arabidopsis*. Plant Cell.

[CR50] Nakanishi H, Yamaguchi H, Sasakuma T, Nishizawa NK, Mori S (2000). Two dioxygenase genes, *Ids3* and *Ids2*, from *Hordeum vulgare* are involved in the biosynthesis of mugineic acid family phytosiderophores. Plant Mol Biol.

[CR51] Negishi T, Nakanishi H, Yazaki J, Kishimoto N, Fujii F, Shimbo K, Yamamoto K, Sakata K, Sasaki T, Kikuchi S, Mori S, Nishizawa NK (2002). cDNA microarray analysis of gene expression during Fe-deficiency stress in barley suggests that polar transport of vesicles is implicated in phytosiderophore secretion in Fe-deficient barley roots. Plant J.

[CR52] Nishiyama R, Kato M, Nagata S, Yanagisawa S, Yoneyama T (2012). Identification of Zn-nicotianamine and Fe-2′-deoxymugineic acid in the phloem sap from rice plants (*Oryza sativa* L.). Plant Cell Physiol.

[CR53] Nishizawa N, Mori S (1987). The particular vesicle appearing in barley root cells and its relation to mugineic acid secretion. J Plant Nutr.

[CR54] Nozoye T, Itai RN, Nagasaka S, Takahashi M, Nakanishi H, Mori S, Nishizawa NK (2004). Diurnal changes in the expression of genes that participate in phytosiderophore synthesis in rice. Soil Sci Plant Nutr.

[CR55] Nozoye T, Nagasaka S, Kobayashi T, Takahashi M, Sato Y, Sato Y, Uozumi N, Nakanishi H, Nishizawa NK (2011). Phytosiderophore efflux transporters are crucial for iron acquisition in graminaceous plants. J Biol Chem.

[CR56] Nozoye T, Nagasaka S, Bashir K, Takahashi M, Kobayashi T, Nakanishi H, Nishizawa NK (2014). Nicotianamine synthase 2 localizes to the vesicles of iron-deficient rice roots, and its mutation in the YXXφ or LL motif causes the disruption of vesicle formation or movement in rice. Plant J.

[CR57] Ogo Y, Itai RN, Nakanishi H, Inoue H, Kobayashi T, Suzuki M, Takahashi M, Mori S, Nishizawa NK (2006). Isolation and characterization of IRO2, a novel iron-regulated bHLH transcription factor in graminaceous plants. J Exp Bot.

[CR58] Ogo Y, Itai RN, Nakanishi H, Kobayashi T, Takahashi M, Mori S, Nishizawa NK (2007). The rice bHLH protein OsIRO2 is an essential regulator of the genes involved in Fe uptake under Fe-deficient conditions. Plant J.

[CR59] Ogo Y, Kobayashi T, Itai RN, Nakanishi H, Kakei Y, Takahashi M, Toki S, Mori S, Nishizawa NK (2008). A novel NAC transcription factor IDEF2 that recognizes the iron deficiency-responsive element 2 regulates the genes involved in iron homeostasis in plants. J Biol Chem.

[CR60] Ogo Y, Itai RN, Kobayashi T, Aung MS, Nakanishi H, Nishizawa NK (2011). OsIRO2 is responsible for iron utilization in rice and improves growth and yield in calcareous soil. Plant Mol Biol.

[CR61] Ogo Y, Kakei Y, Itai RN, Kobayashi T, Nakanishi H, Takahashi H, Nakazono M, Nishizawa NK (2014). Spatial transcriptomes of iron-deficient and cadmium-stressed rice. New Phytol.

[CR62] Qi YH, Wang SK, Shen CJ, Zhang SN, Chen Y, Xu YX, Liu Y, Wu YR, Jiang DA (2011). OsARF12, a transcription activator on auxin response gene, regulates root elongation and affects iron accumulation in rice (*Oryza sativa*). New Phytol.

[CR63] Rellán-Álvarez R, Giner-Martínez-Sierra J, Orduna J, Orera I, Rodríguez-Castrillón JA, García-Alonso GI, Abadía J, Álvarez-Fernández A (2010). Identification of a tri-iron(III), tri-citrate complex in the xylem sap of iron-deficient tomato resupplied with iron: new insights into plant iron long-distance transport. Plant Cell Physiol.

[CR64] Rodríguez-Celma J, Lin WD, Fu GM, Abadía J, López-Millán AF, Schmidt W (2013). Mutually exclusive alterations in secondary metabolism are critical for the uptake of insoluble iron compounds by Arabidopsis and *Medicago truncatula*. Plant Physiol.

[CR65] Römheld V, Marschner H (1986). Evidence for a specific uptake system for iron phytosiderophore in roots of grasses. Plant Physiol.

[CR66] Sasaki A, Yamaji N, Yokosho K, Ma JF (2012). Nramp5 is a major transporter responsible for manganese and cadmium uptake in rice. Plant Cell.

[CR67] Schmid NB, Giehl RFH, Döll S, Mock HP, Strehmel N, Scheel D, Kong X, Hider RC, von Wirén N (2014). Feruloyl-CoA 6′-hydroxylase1-dependent coumarins mediate iron acquisition from alkaline substrates in Arabidopsis. Plant Physiol.

[CR68] Shojima S, Nishizawa NK, Fushiya S, Nozoe S, Kumashiro T, Nagata T, Ohata T, Mori S (1989). Biosynthesis of nicotianamine in the suspension-cultured cells of tobacco (*Nicotiana megalosiphon*). Bio Metal.

[CR69] Shojima S, Nishizawa NK, Fushiya S, Nozoe S, Irifune T, Mori S (1990). Biosynthesis of phytosiderophores: *in vitro* biosynthesis of 2′-deoxymugineic acid from L-methionine and nicotianamine. Plant Physiol.

[CR70] Takagi S (1976). Naturally occuring iron-chelating compounds in oat- and rice-root washing. I. Activity measurement and preliminary characterization. Soil Sci Plant Nutr.

[CR71] Takagi S, Nomoto K, Takemoto S (1984). Physiological aspect of mugineic acid, a possible phytosiderophore of graminaceous plants. J Plant Nutr.

[CR72] Takahashi M, Yamaguchi H, Nakanishi H, Shioiri T, Nishizawa NK, Mori S (1999). Cloning two genes for nicotianamine aminotransferase, a critical enzyme in iron acquisition (Strategy II) in graminaceous plants. Plant Physiol.

[CR73] Takahashi R, Ishimaru Y, Senoura T, Shimo H, Ishikawa S, Arao T, Nakanishi H, Nishizawa NK (2011). The OsNRAMP1 iron transporter is involved in Cd accumulation in rice. J Exp Bot.

[CR74] Wang L, Ying Y, Narsai R, Ye L, Zheng L, Tian J, Whelan J, Shou H (2013). Identification of OsbHLH133 as a regulator of iron distribution between roots and shoots in *Oryza sativa*. Plant Cell Environ.

[CR75] Wu J, Wang C, Zheng L, Wang L, Chen Y, Whelan J, Shou H (2011). Ethylene is involved in the regulation of iron homeostasis by regulating the expression of iron-acquisition-related genes in *Oryza sativa*. J Exp Bot.

[CR76] Yang A, Li Y, Xu Y, Zhang WH (2013). A receptor-like protein RMC is involved in regulation of iron acquisition in rice. J Exp Bot.

[CR77] Yokosho K, Yamaji N, Ueno D, Mitani N, Ma JF (2009). OsFRDL1 is a citrate transporter required for efficient translocation of iron in rice. Plant Physiol.

[CR78] Yoshii M, Yamazaki M, Rakwal R, Kishi-Kaboshi M, Miyao A, Hirochika H (2010). The NAC transcription factor RIM1 of rice is a new regulator of jasmonate signaling. Plant J.

[CR79] Yoshino M, Murakami K (1998). Interaction of iron with polyphenolic compounds: application to antioxidant characterization. Anal Biochem.

[CR80] Zhang Y, Xu YH, Yi HY, Gong JM (2012). Vacuolar membrane transporters OsVIT1 and OsVIT2 modulate iron translocation between flag leaves and seeds in rice. Plant J.

[CR81] Zhang L, Itai RN, Yamakawa T, Nakanishi H, Nishizawa NK, Kobayashi T (2014). The Bowman-Birk trypsin inhibitor IBP1 interacts with and prevents degradation of IDEF1 in rice. Plant Mol Biol Rep.

[CR82] Zheng L, Ying Y, Wang L, Wang F, Whelan J, Shou H (2010). Identification of a novel iron regulated basic helix-loop-helix protein involved in Fe homeostasis in *Oryza sativa*. BMC Plant Biol.

[CR83] Zheng L, Yamaji N, Yokosho K, Ma JF (2012). YSL16 is a phloem-localized transporter of the copper-nicotianamine complex that is responsible for copper distribution in rice. Plant Cell.

