# Supplementary figures and images for: Iron deficiency responses in rice roots
Source: Rice (N Y). 2014 Oct 7;7:27. doi: 10.1186/s12284-014-0027-0 (PMC4884003; doi:10.1186/s12284-014-0027-0)

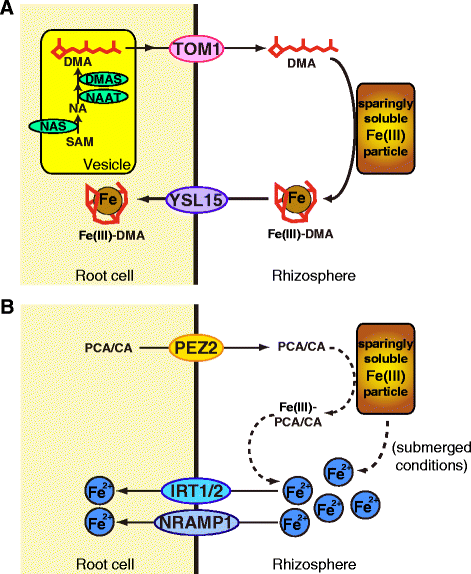

Supplement: Supplementary file 1 — Authors’ original file for figure 1 [file 12284_2014_27_MOESM1_ESM.gif]

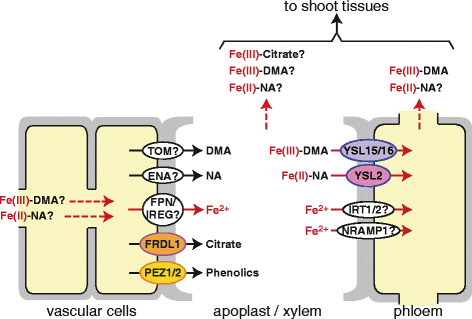

Supplement: Supplementary file 2 — Authors’ original file for figure 2 [file 12284_2014_27_MOESM2_ESM.gif]

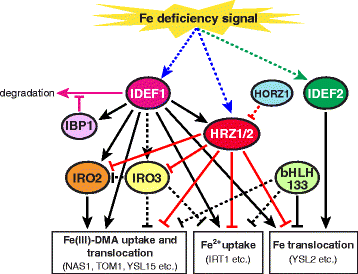

Supplement: Supplementary file 3 — Authors’ original file for figure 3 [file 12284_2014_27_MOESM3_ESM.gif]
